# Implementation of Near-Peer Learning for the Sustainability of Rural Family Medicine Education

**DOI:** 10.7759/cureus.43709

**Published:** 2023-08-18

**Authors:** Nozomi Nishikura, Ryuichi Ohta, Chiaki Sano

**Affiliations:** 1 Community Care, Unnan City Hospital, Unnan, JPN; 2 Community Medicine Management, Shimane University, Izumo, JPN

**Keywords:** near peer learning, general medicine, patient care, medical education, rural japan, family medicine

## Abstract

Balancing educators and trainees in community-based medical education (CBME) is essential for practical education in family medicine and the quality of care. The number of educators and trainees can be flexible in rural family medicine education. Implementing near-peer learning (NPL), in which trainees learn from each other and enhance their clinical skills, is complementary to medical education in rural medical education, which lacks medical educators. The Department of Community Care at the Unnan City Hospital has experienced significant changes in staffing structure. The previous structure of two consultants and six senior residents was replaced by losing one consultant and adding three senior residents. Therefore, the balance between the numbers of educators and learners changed significantly. Traditional teamwork methods no longer ensure effective team communication and balance; currently, effective teamwork does not occur within a team. The increased burden on consultants could result in lower patient outcomes and decreased quality of education for students and residents, thereby affecting the nurturing of future generations. To overcome these difficulties, we implemented the NPL. The implementation was based on strengths, weaknesses, opportunities, and threats (SWOT) and stakeholder analyses. This technical report demonstrated that NPL in rural family medicine education benefits the quality of rural medical education.

## Introduction

Balancing educators and trainees in community-based medical education (CBME) is essential for practical education in family medicine and the quality of care. CBME is a practical educational method through which medical trainees learn about primary care and family medicine in institutions other than universities and tertiary hospitals [1,2]. The number of educators and trainees can be flexible in rural family medicine education, and the implementation of near-peer learning (NPL), in which trainees learn from each other and enhance their clinical skills, complements medical education in rural medical education, which lacks medical educators [3].

Patient management and education are critical to patient outcomes in rural family medicine CBMEs, and patient management is directly related to patient outcomes. The lack of balance between consultants and residents may decrease the percentage of time devoted to education by consultants because they need to focus more on patient management [4]. This can lead to a decline in the quality of residency programs, making them less attractive to students and residents who are future candidates for family medicine specialization. In the long term, this may jeopardize the Department of Family Medicine.

The Department of Community Care at the Unnan City Hospital experienced significant changes in its staffing structure. The increased burden on consultants could result in lower patient outcomes and decreased quality of education for students and residents, thereby affecting the nurturing of future generations. To overcome these difficulties, we implemented the NPL. The implementation was based on strengths, weaknesses, opportunities, and threats (SWOT) and stakeholder analyses. This technical report shows that NPL in rural family medicine education benefits rural medical education.

## Technical report

Context

The Unnan City Hospital is a community hospital located in one of the most remote areas in Japan and implements CBME. The Department of Community Care was established in 2016 at the Unnan City Hospital with the vision of enabling citizens to lead healthy lives and live as they are until the end of their lives. Our department’s mission is to enrich comprehensive care by providing community care in the city of Unnan. The critical aspect of this mission is essential for attracting medical students and residents to work at the Unnan City Hospital. Accompanied by an established educational system for family medicine, the department collects information from several medical residents of family medicine to revise the educational system for a better quality of education and patient care [5].

Each team member plays a different role in the community care department. The consultants manage their outpatients and ward patients, supervise and educate each senior resident, and teach students and junior residents. Senior residents develop their competencies as family physicians mainly by managing outpatients, emergency departments, and ward patients at our hospital and through home care and clinics outside our hospital. Second-year junior residents from tertiary hospitals and fifth- and sixth-year medical school students undergo family medicine training. Recruiting them to become interested in family medicine and encouraging them to join our department is essential for our staffing because this hospital lacks an educational program for junior medical residents [4].

**Figure 1:**
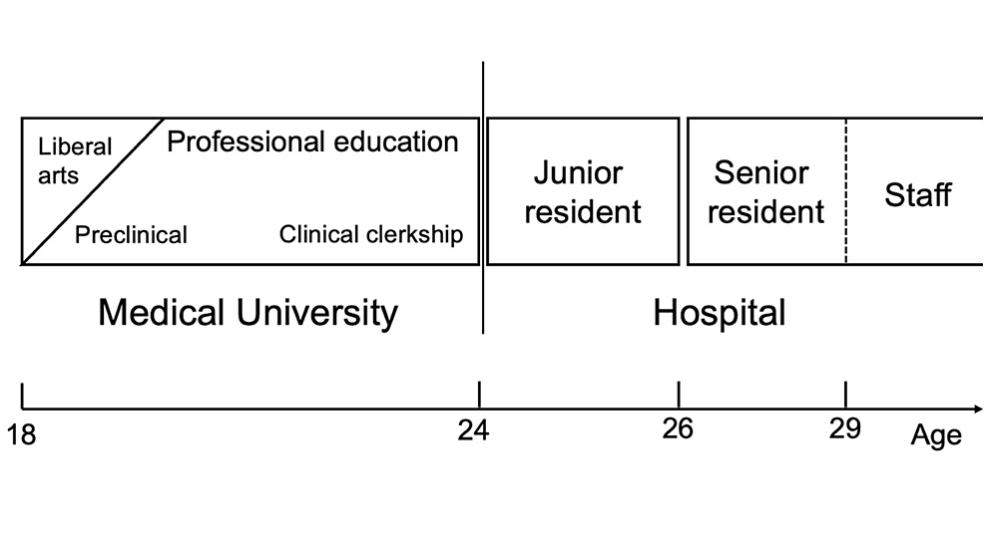
Overview of clinical training in Japan In Japan, physicians must complete two years of training at a designated hospital after graduating from university. Doctors in their first two years of training are junior residents, and those who obtain specialist certification are senior residents.

At the time of foundation, the department had two consultants. Since then, we have increased the number of senior residents by recruiting them into senior residency programs to expand the department’s activities. By 2021, there were two medical advisors and six residents, and the role of educators was shared with two consultants. The composition of consultants as educators and senior residents as learners and frontline workers allowed us to divide these roles without problems (Figure 1).

**Figure 2:**
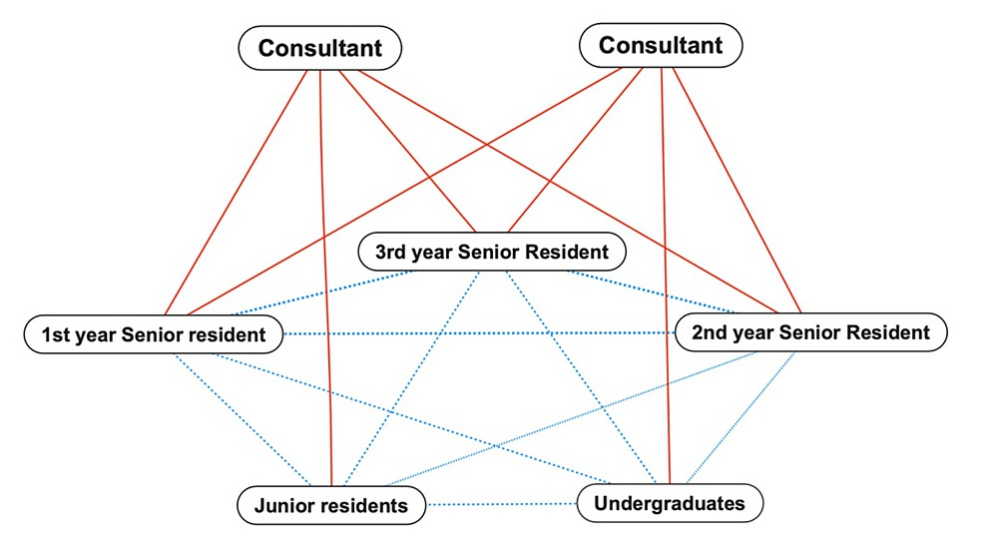
Previous team collaboration

We welcomed three new residents in April 2022, and the Department of Community Care entered a new phase with the departure of one of the consultants, a founding department member. This change disrupted the previously well-balanced structure between educators and learners. Using the traditional structural format and restricting the educator’s role to the consultant could cause two potential issues: a decline in patient outcomes due to a decrease in patient management skills and a drop in the quality of family medicine programs that attract medical students and residents. Therefore, the impact of changing the consultant-to-resident ratio from 2:6 to 1:9 should be approached by revising the department’s management and educational systems (Figure 2).

NPL theory

NPL theory was implemented in our team management. NPL is a theoretical framework emphasizing the significance of a similar knowledge base between teachers and learners and mutual learning among nonprofessional teachers and learners in groups [6]. NPL can alleviate the pressure on overburdened medical teachers and preserve the quality of medical education in situations with limited medical and educational resources [7]. Therefore, NPL would fit well within our department’s limited educational resources. It can enhance residents’ professional self-efficacy and increase job satisfaction through mutual education during training [8]. Additionally, monitoring mentors and learners increases residents’ clinical competence and professionalism [9,10].

**Figure 3:**
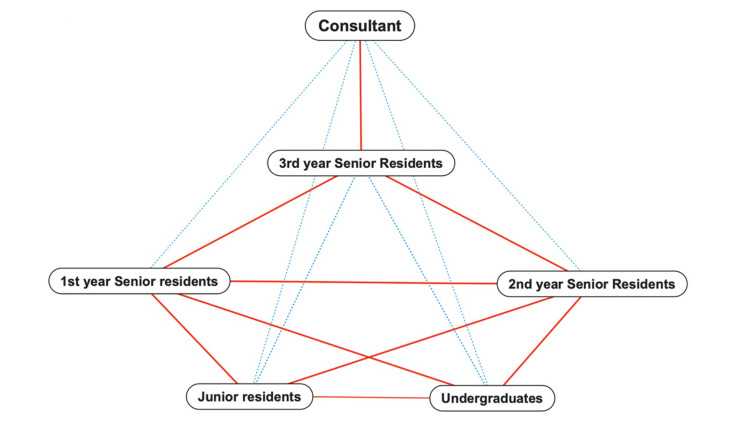
Revised team collaboration

We implemented the NPL in our department by clarifying each team member's role. This process was a process improvement program implementation. First- and second-year senior residents were responsible for patient management and educating medical students and junior residents. Additionally, they were instructed to accompany students/junior residents to senior residents’ outpatient units and wards as much as possible and reflect on what students/junior residents had learned. Third-year residents were responsible for consulting and providing appropriate feedback to first- and second-year residents and for the overall management of senior residents. Third-year residents were also liaised between supervising the physicians and second-year residents. The consultant will continue to manage the entire residency while discussing patient management with the third-year residents. Additionally, as the burden of managing the program decreased, supervisors reviewed each team member to support their learning. The solid red lines indicate strong connections, and the dotted blue lines indicate weak connections. Before implementation, the structure was consultant-versus-resident. After implementation, the structure was hierarchical from consultants to third-year, first- and second-year senior residents, and finally to students and junior residents (Figure 3).

Organizational and stakeholder analysis

We analyzed our department’s situation using a SWOT analysis to determine our preparedness for the NPL introduction and what needed our attention (Table 1) [11].

**Table 1:** SWOT analysis

	Strengths	Weakness
Opportunities	The history of our department has increased the tolerance for innovation. The training of general practitioners is being encouraged at the national level, making it easier for students who come to our hospital to become general practitioners and join our department in the future.	A trend toward training general practitioners is emerging; however, the number of consultants is insufficient to provide education.
Threats	Japan has a culture that values a vertical society, but senior residents are close to each other, which may make communication easier. Residents had little opportunity to take on the role of an educator. However, our hospital has a supportive environment with medical education specialists.	Japan has a culture that values a vertical society, and residents are not accustomed to expressing their opinions in discussions. Residents’ workload is heavy, and the time available for education is shortened due to the reform of the Japanese work style.

A SWOT analysis was performed based on a questionnaire administered to eight team members regarding four points: team strengths, weaknesses, opportunities, and threats to the Department of Community Care. A SWOT analysis was performed through a discussion of the NN and RO.

Based on the SWOT analysis, our educational reform could significantly impact the department’s team members more than other stakeholders, require their cooperation in the change, and enlist their help in analyzing the current situation. For effective reform, we used a stakeholder table to analyze the interaction between each stakeholder and the reform (Table 2) [12].

**Table 2:** Stakeholder analysis CBME: community-based medical education

Stakeholder name	Impact	Influence	What is important to the stakeholder?	How could the stakeholder contribute to the project?	How could the stakeholder block the project?	Strategy for engaging the stakeholder
	Low, medium, high	Low, medium, high				
Consultant	High	High	Maintain patient outcomes. Supporting the effective growth of the team’s personnel.	Observation and management of the entire team. Coordination of systems in close collaboration with third-year residents. Regulation of relationships with stakeholders outside of the department.	The new structure cannot be maintained by taking consultations from senior residents as before.	Can discuss with consultants and get advice any time.
Third-year residents	High	High	Improvement of own patient management skills. Acquiring a role as an educator. Acquiring team management skills.	Communicate with the consultant to coordinate systems. Deepen the connection by communicating more with other senior residents.	Possibility of decreased patient outcomes by making decisions beyond their own patient management capacity. Possibility of not being able to communicate closely with consultants and other residents when they become too busy with their own work.	Explain to them how it works, what is good about this system, and what should be of concern. Create an environment that allows immediate feedback to the consultant if they have any problems.
First- and second-year senior residents	High	High	Improvement of own patient management skills. Acquiring a role as an educator.	Consult with third-year residents first. Work closely with students and junior residents.	Possibility of being unable to communicate closely with third-year residents and students when they become too busy with their work. Possibility of seeking help from the consultant because they feel anxious about consulting with third-year residents when they have problems.	Explain how it works, what is good about this system, and what should be of concern. Ensure they understand that they can communicate with their supervisor anytime.
Junior residents, medical students	Medium	Medium	Learn community healthcare in our CBME program.	Increase educator competency of first- and second-year senior residents by asking them questions.		Embed the NPL into the program as a rule of practice.
Patients	Medium	Low	Maintain patients’ outcomes.	Tolerance and acceptance of examinations by students and junior residents.	Strong preference for seeing a consultant and refusal to accept residents or students.	Explain that the hospital is a teaching hospital for students and residents and obtain their consent.
Other healthcare professionals	Medium	Medium	Smoothly manage patients.		Immediately seek the opinion of the consultant to expect a prompt response.	Demonstrate that there is an adequate support system for the consultant. Show that there are no delays in patient management during implementation.
Other doctors in our hospital	Low	Low	Good collaboration with our department in patient management.		Immediately seek the opinion of the consultant to expect a prompt response.	Demonstrate that there is an adequate support system for the consultant. Show that there are no delays in patient management during implementation.

## Discussion

There are several points of concern when implementing NPL in family medicine training and patient management. We discussed this by stratifying the contents of the SWOT analysis from an ecological-theoretical perspective.

Considering Japanese culture as a macrosystem element, emphasizing vertical relationships may have advantages and disadvantages. For example, in the trainer-trainee relationship between the consultant and residents, the consultant’s opinion is somehow unconsciously followed [13]. However, the introduction of NPL may lead to constructive discussions because the relationships between trainers and trainees are less vertical in NPL [14]. The trainees are not accustomed to expressing their opinions during the discussions. However, with only nine residents in this department, they knew each other well, and communication was smoother than in a department with many residents, such as a university hospital.

From an ecosystem perspective, senior residents have not been trained as educators in junior residency programs. In Japan, the role of the educator is not specified in the competencies of junior residency programs; in fact, there is little experience regarding this role. However, the consultant is an expert in medical education, and one of our third-year senior residents specializes in medical education [15]. The presence of educational professionals is advantageous in implementing NPL. Reflecting on senior residents will enhance their competency as educators.

Considering the environment of our hospital, one barrier is the heavy workload of the senior residents. Therefore, third-year residents must understand the situation of senior trainees through reflection and adjust their balance through constant discussions with consultants [16]. Finally, it is essential to consider the unique nature of the COVID-19 pandemic. Medical personnel always live alongside the possibility of infection [17]. Regarding risk management, it would be advantageous for this emergent situation that multiple physicians understand patients' conditions, and the patient's information is shared with multiple members through the NPL.

## Conclusions

CBME is crucial in training future family medicine practitioners, especially in rural settings like Unnan City Hospital. The balance between educators and trainees is pivotal for maintaining educational quality and patient outcomes. When the consultant-to-resident ratio shifted unfavorably, the introduction of NPL became a strategic response. This method leverages shared knowledge between closely aligned roles, alleviating strain on primary educators and bolstering the educational experience. Factors such as Japan's traditionally vertical societal structure posed challenges and benefits to NPL's implementation. While hierarchy can sometimes stifle discussion, the small department size facilitated smoother communication. A notable obstacle was the inexperience of senior residents in educator roles, given Japan's junior residency programs' emphasis. However, the department's support from medical education experts provided a significant advantage. Implementing NPL, especially with the added complexity of the COVID-19 pandemic, offers a multipronged approach: enriching the educational experience, optimizing patient management, and providing a safety net against potential staff shortages.
